# Trajectories of maternal symptoms of depression and anxiety over 13 years: the influence of stress, social support, and maternal temperament

**DOI:** 10.1186/1471-2458-12-1120

**Published:** 2012-12-27

**Authors:** Anni Skipstein, Harald Janson, Anne Kjeldsen, Wendy Nilsen, Kristin S Mathiesen

**Affiliations:** 1 Norwegian Institute of Public Health, Division of Mental Health, PO Box 4404, Nydalen, Oslo 0403, Norway; 2 The Norwegian Center for Child Behavioral Development, PO Box 7053, Majorstuen, Oslo 0306, Norway

**Keywords:** Symptoms of depression and anxiety, Trajectories, Temperament, Stressors, Social support

## Abstract

**Background:**

Depression and anxiety are the most common mental health problems among women, with various negative impacts both for the women concerned and their families. Greater understanding of developmental trajectories of maternal symptoms of depression and anxiety over the child rearing period would have significant benefits for public health, informing prevention and treatment approaches. The aim of the current study was to examine whether stressors related to child rearing and living conditions, social support, and maternal temperament, predicted mothers’ membership in groups with different trajectories of symptoms of depression and anxiety during 13 years of the child rearing phase.

**Methods:**

The data were from a prospective, longitudinal study of 913 mothers in Norway followed from when their children were 18 months old (time 1) until they were 14.5 years (time 6) (the TOPP study). Multinomial logistic regression analyses were used to test whether child related stressors, stressors related to the living conditions, social support and maternal temperament at time 1 predicted membership in groups based on maternal symptoms of depression and anxiety over the subsequent 13 years.

**Results:**

Temperamental distress, followed by child related stressors, were the strongest predictors of membership in a group with high symptoms of depression and anxiety over time. Stressors related to living conditions, and social support from partner and friends/family were also significant predictors. No interaction effects among predictors were found.

**Conclusions:**

This study indicates that factors present early in the child rearing phase may provide substantial prediction of the variance in maternal symptoms of depression and anxiety over the following 13 years. Temperamental distress and child related stressors were the strongest predictors of membership in different depression and anxiety symptom trajectory groups.

## Background

Depression and anxiety are the most common mental health problems among women [1,2]. These problems can have adverse consequences for individual women, their families and society as a whole. The personal costs are huge – with reduced life quality and functioning with regards to relationships with partners and children, friends, and work life [3-5]. Maternal depression is an important risk factor for child psychopathology [6], and remission of maternal depression is related to improved child outcomes [7]. However, the majority of studies focusing on maternal depression have been on postpartum depression [6]. Therefore, little is known about the persistence of elevated depressive symptoms in mothers from their children were toddlers and throughout the teenage period [8,9]. Hence exploring predictors of maternal depressive symptoms throughout the child rearing period is important, with significant public health benefits from better knowledge to inform prevention efforts.

Typically depression is explained by a diathesis-stress model in which stressors play a triggering role in depression, affecting the timing, and possibly the severity and duration, of symptoms through various cognitive, neurobiological, and social vulnerability mechanisms [10,11]. There is considerable evidence of the importance of stress in the development of symptoms of depression and anxiety [10,12]. The research on the relative effects of different sources of stress on symptoms of depression is, however, sparse. This is especially true for effects of chronic stress factors [11]. Some findings indicate that chronic stressors are associated with depression more strongly and over a longer period than acute stressors [13-15]. It is therefore important to assess the relative effects of chronic stress factors from different sources when examining the role of stressors on symptoms of depression and anxiety.

### Child rearing period

Motherhood can be accompanied by a variety of chronic stressors that may contribute directly or indirectly to depression [16,17]. Whereas several studies have examined the effects of parental depression on parenting and on children’s emotional and behaviour problems, few studies have examined the reverse pattern; that is, how stressors related to having children and parenting may influence maternal depression trajectories [4].

Stressors related to other living conditions (such as housing problems, work difficulties, and partner’s health problems) are associated with depression and anxiety [16,18-20]. Lack of social support has been found to have a direct effect on depression among mothers with young children [16]. However, few studies have followed mothers and their offspring from infancy to adolescence to examine how stressors related to their children and living conditions might influence their levels of depression and anxiety across a longer time period.

One of the few studies well suited to examine the role of stressors related to children and living conditions on maternal mental health across time is the Norwegian TOPP study (‘Tracking Opportunities and Problems Project’). The TOPP study, following a community based sample of 18-month old toddlers and their families, has shown direct effects of early child related stressors on maternal symptoms of depression and anxiety when the children were of preschool age [18-20]. The same dataset is utilized in the current study, focusing on early predictors of longer term development of symptoms of depression and anxiety amongst mothers.

### Early stressors and symptom trajectories

Rearing young children requires considerable social, financial, and health care resources. Psychological distress is likely to result if resources are scarce; for example, symptoms of depression and anxiety are more prevalent among persons with lower compared to higher socio-economic status [21,22], and evidence indicates that both socio-economic disadvantage and absence of social support are associated with maternal symptoms of depression and anxiety [16,23]. Lower maternal age, fewer years of education and lack of work participation have been found to relate to maternal symptoms of depression and anxiety in the TOPP study [24]. Financial stress increases the risk of poor maternal health, and mothers who report multiple stressors are at particularly high risk [23].

It is also possible that stressors related to children and to living conditions interact, thereby increasing the negative effect of early stressors on long term symptom development. A Dutch study found that having a job protected against developing symptoms of depression and anxiety for women without children, but not for women with children [25]. Hence, women with young children might be more susceptible to the stresses of juggling the demands of multiple roles, such as being an employee and a parent. Problems with housing and economy might also be more problematic when there are children to take care of as well.

### Social support

In addition to directly affecting mental health, social relationships can act as a buffer against negative effects of stress on mental health [26,27]. Such effects were demonstrated in a cross-sectional study where mothers who reported high levels of support from their partner experienced less negative emotional impact from various stressors [28]. Other studies, however, report that several of the documented links between perceived support and mental health reflect main effects rather than buffering or moderating the effects of stress [12,29]. The idea that specific types of stress may be buffered by specific types of support has evolved over recent years [27], indicating the importance of studying different sources of stress and support. Having toddlers can be demanding, and receiving good social support in this period may be important for the mother’s sense of coping, and may affect symptom development later in the child rearing phase. Few projects have studied long term effects of child related stressors and whether social support from partners and/or family and friends might buffer the effect of early stressors. Knowledge about the relationship between social support and different sources of stress on maternal long term symptom development can inform preventive strategies.

### Temperament

Temperament is known to influence depression and anxiety [30]. Temperament refers to individual differences in basic styles of behaviour such as emotionality, activity level and sociability [31]. Research on adults has tended to examine personality rather than temperament, although these two terms are often used interchangeably [30] and are closely interrelated. For example, temperamental emotionality is empirically and theoretically linked to the personality construct of neuroticism/negative emotionality [32], high levels of which appear to precede the onset of depression [30,32]. Some studies have found direct effects of personality on depression, whilst others have found interaction effects between life stress and personality [12]. In a review of stress and depression, Hammen (2005) found that in some studies neuroticism moderated the effect of stress on depression, and suggested that more research in this area is needed. She also noted that neuroticism might be a genetically transmitted predisposition for both stressful events and depression [33]. Personality or temperament factors may therefore be moderators of the association between stressors and symptoms of depression and anxiety.

The overall aim of the current study was to identify predictors assessed early in the child-rearing period (when children were 18 months of age) that discriminated between groups of mothers with different trajectories of depression and anxiety symptoms over the subsequent 13 years (up to when their children were 14.5 years of age). It built upon a previous study using the same dataset that identified six maternal symptom trajectories with six waves of data over this time period [24]. Using subgroups of mothers with different trajectories of symptoms of depression and anxiety might provide knowledge about whether there are different predictors for groups of mothers with different symptom severity and persistence.

Specifically, the aim of the study was to examine whether stressors related to child rearing and living conditions, social support (from partners and/or from friends and family), and maternal temperament, as assessed when women were caring for 18 month old children, predicted mothers’ membership in groups with different trajectories of symptom development during the following 13 years and whether there were interaction effects between the various stressors. In addition, we hypothesized that child related stressors and stressors related to living conditions might have a stronger effect on symptoms of depression and anxiety among mothers with lower social support, and that temperament may be a moderator of the association between stressors and symptoms of depression and anxiety.

## Methods

### Sample and procedure

The current study used data from the TOPP study, a population-based prospective longitudinal study focusing on developmental trajectories to well-being and symptoms of mental health problems among children, adolescents and their parents in Norway. The longitudinal data collection has been approved by The Data Inspectorate and the Regional Committee for Medical Research Ethics. The TOPP study is subject to the ethical guidelines and rules of confidentiality that apply in the National Committee for Research Ethics in the Social Sciences and the Humanities.

More than 95% of all families in Norway with small children regularly attend a child health clinic during the early childhood years. All families from 19 health care areas in eastern Norway who visited a child health clinic in 1993 for their child’s scheduled 18 months visit were invited to complete a questionnaire and participate in the TOPP study.

Of 1081 eligible families at the child health clinic, 913 mothers participated at time 1 (t1). At time 2 when the child was 2.5 years old, 777 mothers (85% of t1) participated. At time 3, when the child was 4.5 years, 727 (80% of t1) mothers participated. At t4, when the child was 8.5 years, 505 (55% of t1) mothers participated. At time 5, when the child was 12.5 years, 587 (64% of t1) mothers participated, and at time 6, when the child was 14.5 years, 474 mothers (52% of t1) participated. The questionnaires were administered at the child health clinics for the first three time points and by post from t4. More than 95% of the mothers were Norwegian born.

#### Representativeness and attrition

Background information on the mothers who declined to respond at t1 was recorded at the child health clinic. Non-respondents did not differ significantly from respondents with respect to maternal age, education, employment status, number of children, and marital status.

Attrition analyses showed no significant differences between the mothers who filled in questionnaires at all waves and those who participated only in the first wave with respect to age, number of children, financial status, social support, chronic stress, temperament, negative life events, and maternal symptom level. A study based on the same data set found that the only significant predictor of drop out from the study was maternal education [34]. In addition, after performing a Monte Carlo simulation study, they concluded that estimates of correlations involving socio-economic factors may be under-estimated while estimates of correlations between psychological and relationship variables seem to be valid even at attrition rates up to 50% [34].

#### Child related stressors

At t1, mothers were asked to indicate whether they had experienced ongoing problems during the last 12 months in the following areas: a) Problems with child care arrangements (e.g. day care, babysitter, or when the child is ill); b) Problems combining work and family responsibilities; c) Problems concerning the child’s health (e.g. disability or illness); and d) Problems with upbringing, supervision, school, and discipline. These items were to be answered in relation to all their children, not only the target child aged 18 months, with response categories from 1 (none), to 4 (very large problems). A scale of child related stressors was constructed by computing the average of these four items. Cronbach’s Alpha was .57 for this scale.

#### Stressors related to living conditions

The same introduction and response categories as for child related stressors above was used to assess three sources of stress related to living conditions at t1: a) Housing problems (maintenance, rental agreement etc.); b) Employment (unemployment, uncertain work, difficult work relations); and c) Economic problems. A scale of living condition stressors was constructed by computing the average of these three items. Cronbach’s Alpha was .62 for this scale.

#### Social support from partner

To measure social support from partners, three items about emotional support from the mothers’ romantic partner were used (“I feel closely attached to my partner”, “My partner put cares about my opinions”, “Sometimes I feel excluded, even in my own home”), each rated on a Likert scale from 1 (completely disagree) to 5 (completely agree). The three items were averaged to construct a scale of partner support, with a Cronbach’s Alpha of .59.

#### Social support from friends and family

To measure social support from friends and family, we used three items tapping emotional support and feeling of belonging from friends and three items about support from the family (“I feel closely attached to my family/friends”, “My family/friends cares about my opinions”, “Sometimes I feel excluded, even among my friends/family”). These six items, each rated on a Likert scale from 1 (completely disagree) to 5 (completely agree), were averaged to construct a scale of social support from friends and family, with a Cronbach’s Alpha of .75.

#### Temperament

Maternal temperament was measured with a Norwegian translation of the Emotionality, Activity and Sociability scale (EAS) for adults [35]. This is a 20-item self report instrument consisting of five subscales: Activity; Sociability; Distress; Fearfulness; and Anger. The latter three, which together measure the broader construct of the temperament trait emotionality, were used in the current study. The items (e.g. “I get emotionally upset easily”, “I often feel insecure”, “There are many things that annoy me”) were scored on a Likert scale from 1 (not typical) to 5 (very typical). A score for each temperament subscale was constructed by computing the average of its four items. The factor structure and psychometric properties of the EAS scale have previously been assessed in this sample and shown to be satisfactory. Test-retest correlations suggested that the Alpha values slightly underestimated the reliability (see Naerde, Roysamb & Tambs, 2004 for more) [36]. Cronbach’s Alphas at t1 for the temperament subscales Distress, Fearfulness and Anger were .73, .57 and .58, respectively.

#### Socio-demographic variables

Maternal age was computed from mothers’ report of their year of birth. Maternal education was coded as number of years of completed education. Self-report of workforce participation was coded as either having or not having paid work.

#### Symptoms of depression and anxiety

Symptoms of depression and anxiety were measured by a Norwegian version of the Hopkins Symptom Check List (HSCL-25) [37]. The original checklist consists of 25 items creating two highly correlated scales for depression (15 items) and anxiety (10 items). Respondents indicate the degree of being burdened by each symptom on a four-point Likert scale ranging from 1 (not at all) to 4 (extremely). The average score across all items was used as the index of maternal symptoms of depression and anxiety. The HSCL version used in the current study comprised 23 items at t1 and t2, 24 items at t3, t4 and t6, and 10 items at t5. A pilot study led to two of the original 25 items (“thoughts of ending you life”; “loss of sexual interest or pleasure”) being excluded at t1 and t2 because some mothers perceived them to be offensive. From t3, the first of these items was reintroduced. At the fifth wave the HSCL scale was reduced to 10 items in order to have a shorter questionnaire in a quest for a higher response rate. A study comparing various short versions of the Symptom Checklist found all versions to show almost equally high internal consistency, sensitivity and specificity [38]. Internal consistency (Cronbach’s Alpha) for the HSCL scale at the six time points was high, ranging from .87 to .91. The mean symptom score ranged from 1.28 to 1.41. The temporal stability of HSCL scores ranged from Pearson’s *r* = .52 between t4 and t5 to Pearson’s *r* = .67 between t1 and t2, indicating a high degree of stability of symptom levels [39].

Skipstein and colleagues (2010) identified six profiles, or trajectories, of maternal symptoms of depression and anxiety based on HSCL scores from t1 to t6 using latent profile analysis (LPA). LPA is a person-centred approach designed to divide the population under study into a set of latent subpopulations with similar developmental trajectories [40]. Mplus (version 5) was used to compute trajectories. Skipstein and colleagues (2010) used posterior class membership probabilities estimated after model estimation to assign individuals to pseudo-classes according to the maximum probability rule [24]. The current study also used symptom groups based on the pseudo class membership.

The analysis identified six trajectory groups: 1) a ‘No symptoms’ group with close to zero scores across all waves (5% of the mothers); 2) a ‘Low’ group with consistently low symptom scores across time (19%); 3) a ‘Moderate-low’ group (30%); 4) a ‘Moderate’ group with moderate symptom scores at all waves (32%); 5) a ‘High’ group with overall high symptom levels (10%); and 6) a ‘Low-Rising’ group with initially low symptom levels that increased to a level similar to the High group by t6 (4%) (see Skipstein et al., 2010, for further details).

**Figure 1:**
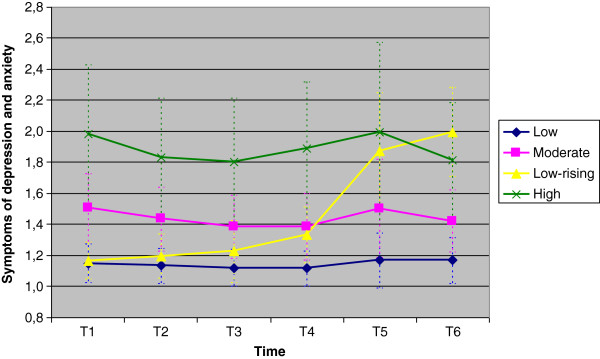
Mean symptom scores and standard deviations (HSCL) in the 4 trajectory groups on each of the 6 waves of data collection.

The three lowest trajectory groups (‘No symptom’, ‘Low’ and ‘Moderate-low’ groups) did not differ significantly on any socio-demographic variables. We decided to merge these three groups into one Low group as we felt this made sense conceptually and made the results easier to portray and interpret. Even though the LPA analysis found differences between the three lowest groups, we were interested in maternal symptom level of a certain magnitude, and in this context making fine distinctions among profiles with low symptom levels was not of interest. Figure 1 shows the means and standard deviations for the HSCL scores of the resulting four symptom trajectory groups at each time point.

### Statistical analyses

Multinomial logistic analyses with SPSS version 17 were used to assess the association of predictor variables with trajectory groups. The continuous variables were standardised before they were entered into the analyses. Prior to the main analyses, a set of exploratory analyses was conducted. In addition to the predictors reported here, sibling status was also included as a predictor in these exploratory analyses. Sibling status did not show any significant effect on maternal symptoms of depression and anxiety. As we also did not have a theoretical rationale to hypothesize an effect of sibling status, this variable was excluded from the analysis in order to reduce complexity of the results. Two sets of analyses were conducted, one with the Low symptom group as reference category and one with the High symptom group as reference category.

## Results

Table 1 shows means and standard deviations of the predictors for the whole sample and for the four symptom groups. The mean levels of both stress indices (i.e. relating to children and to living conditions) were similar, both for the whole sample and across each symptom group.

**Table 1:** Mean values (SD) of predictors for the whole sample and the four trajectory groups

**T1**	**Sample**	**Total**		**Low**		**Low-rising**		**Moderate**		**High**	
	**n**	**Mean**	**(SD)**	**Mean**	**(SD)**	**Mean**	**(SD)**	**Mean**	**(SD)**	**Mean**	**(SD)**
**Maternal age**	913	30.00	(4.72)	30.40	(4.77)	29.80	(4.38)	30.80	(5.16)	28.60	(5.04)
**Stressors**											
Child related	900	1.31	(0.42)	1.20	(0.31)	1.26	(0.32)	1.42	(0.47)	1.58	(0.59)
Living condition	902	1.45	(0.58)	1.29	(0.43)	1.3	(0.37)	1.59	(0.64)	1.91	(0.75)
**Support**											
Partner	805	4.43	(0.73)	4.59	(0.60)	4.68	(0.50)	4.29	(0.76)	3.83	(0.96)
Family/friends	874	4.19	(0.67)	4.35	(0.62)	4.10	(0.68)	4.07	(0.62)	3.72	(0.79)
**Temperament**											
Fearfulness	904	2.24	(0.64)	2.04	(0.53)	2.28	(0.51)	2.45	(0.64)	2.70	(0.77)
Distress	903	2.36	(0.74)	2.04	(0.61)	2.52	(0.74)	2.63	(0.64)	3.14	(0.69)
Anger	903	2.98	(0.70)	2.89	(0.66)	2.85	(0.72)	3.05	(0.72)	3.29	(0.75)
**Years of education**	909	12.70	(2.26)	13.00	(2.23)	12.50	(2.18)	13.30	(2.34)	11.70	(2.31)
**Work participation (%)**	908	63		67		62		65		43	

Mothers in the Low symptom group reported the lowest stress scores and most social support, followed by the mothers in the Low Rising, Moderate and High groups. The mothers reported on average more support from partners than from friends and family. This trend also applied for each of the symptom groups. Mothers with Low-rising symptoms reported the highest levels of partner support, followed by mothers in the Low symptom group. The Low group reported the highest level of support from family and friends.

There was also variation in temperament between the groups. The Low group reported scores well below those of the other three groups. Mothers in the High group reported the highest level of temperamental distress followed by mothers in the Low-rising group. The sample mean for temperamental anger was the highest of the three temperament subscales. Here the Low-rising group reported the lowest level followed by the Low group. The High group had the highest level of temperamental anger.

Table 2 shows results of the multinomial logistic regression analysis with the Low group as reference category. The estimated odds ratios (with 95% confidence intervals) describe the odds of membership in a specific trajectory group relative to the Low group.

**Table 2:** Multinomial regression results with group membership as the dependent variable and the Low symptom group as the reference group

	**Low versus low-rising**		**Low versus moderate**		**Low versus high**	
	**OR**	**95% CI**	**OR**	**95% CI**	**OR**	**95% CI**
**Maternal age**	1.03	(0.67 - 1.57)	0.99	(0.81 - 1.21)	0.97	(0.69 - 1.37)
**Stressors**						
Child related	1.14	(0.66 - 1.96)	1.85***	(1.46 - 2.34)	2.16***	(1.57 - 2.97)
Living condition	0.90	(0.51 - 1.58)	1.49***	(1.21 - 1.84)	1.82***	(1.35 - 2.47)
**Support**						
Partner	1.67	(0.95 - 2.93)	0.88	(0.71 - 1.09)	0.75*	(0.55 - 1.01)
Family/friends	0.62*	(0.42 - 0.93)	0.83	(0.67 - 1.02)	0.60**	(0.45 - 0.84)
**Temperament**						
Fearfulness	1.04	(0.67 - 1.70)	1.54**	(1.21 - 1.95)	1.86**	(1.30 - 2.65)
Distress	3.08***	(1.81 - 5.00)	2.21***	(1,69 - 2,90)	3.53***	(2.28 - 5.49)
Anger	0.71	(0.47 - 1.05)	1.01	(0.83 - 1.23)	1.40*	(1.01 - 1.95)
**Years of education**	1.03	(0.85 - 1.24)	0.89**	(0.81 - 0.97)	0.78**	(0.66 - 0.91)
**Work participation**						
Yes	1.08	(0.46 - 2.52)	1.19	(0.79 - 1.79)	2.06*	(1.05 - 4.07)
No	Reference category					

Note: R^2^ = .40 (Cox & Snell), .45 (Nagelkerke), .25 (McFadden). Model *χ*^2^(30) = 392.78, p < .000; * p < .05; ** p < .01; *** p < .001.

All variables except maternal age were significant predictors of membership in the High symptom group compared to the Low. Child related stressors was the second strongest predictor of membership in the High group compared to the Low, with an odds ratio of 2.16. The odds of being in the High group compared to the Low group also increased with higher levels of stressors related to the living conditions (OR: 1.82). The odds of being in the High group versus the Low also increased with lower levels of support from both partner (OR: 0.75), friends and family (OR: 0.60). In addition the odds increased with higher levels of all of the three sub-scales on temperamental emotionality; distress (OR: 3.53), fearfulness (OR: 1.86), and anger (OR: 1.40).

More years of education reduced the odds of being in the High group compared to the Low group. The odds ratio of being in the Low group versus the High when having paid work was 2.06. Two variables were significant predictors of membership in the Low-rising group compared to the Low group. The strongest predictor was higher levels of temperamental distress (OR: 3.08). The second significant predictor was lower levels of support from friends and family (OR: 0.62).

The odds of being in the Moderate group compared to the Low group increased with higher scores on temperamental fearfulness (OR: 1.54) and distress (OR: 2.21), in addition to having higher levels of both child related (OR: 1.85) and living related stressors (OR: 1.49) when the index child was 18 months old.

The analyses were repeated with the High group as reference category, with a specific focus on the comparison between mothers in the Low-rising versus the High group. Table 3 gives the estimated odds ratios and 95% confidence intervals for the odds of being classified into a trajectory group relative to being classified into the High group.

**Table 3:** Multinomial regression with group membership as the dependent variable and the High symptom group as the reference group

	**High versus low-rising**		**High versus moderate**	
	**OR**	**95% CI**	**OR**	**95% CI**
**Maternal age**	1.06	(0.63 - 1.77)	1.02	(0.74 - 1.40)
**Strain**				
Child related	0.53*	(0.30 - 0.93)	0.86	(0.67 - 1.10)
Living condition	0.50*	(0.27 - 0.90)	0.82	(0.63 - 1,06)
**Support**				
Partner	2.23**	(1.22 - 4.06)	1.17	(0.91 - 1.52)
Family/friends	1.03	(0.65 - 1.65)	1.37*	(1.02 - 1.83)
**Temperament**				
Fearfulness	0.56*	(0.33 - 0.96)	0.83	(0.60 - 1.13)
Distress	0.87	(0.48 - 1.60)	0.63*	(0.42 - 0.93)
Anger	0.51**	(0.31 - 0.82)	0.72*	(0.53 - 0.98)
**Years of education**	1.32*	(1.05 - 1.67)	1.14	(0.99 - 1.33)
**Work participation**				
Yes	0.52	(0.19 - 1.44)	0.58	(0.31 - 1.09)
No	Reference category			

Note: R^2^ = .40 (Cox & Snell), .45 (Nagelkerke), .25 (McFadden).

Model *χ*^2^(30) = 392.78, p < .000; * p < .05; ** p < .01; *** p < .001.

The strongest predictor of the Low-rising group compared to the High group was more support from partner (OR: 2.23), followed by higher levels of education (OR: 1.32). In addition, lower levels of temperamental anger (OR: 0.51), and lower levels of child related stressors (OR: 0.53) at t1 significantly differentiated mothers in the Low-rising group from mothers in the High group. Compared to the High group, being classified into the Moderate group was predicted by lower levels of temperamental distress (OR: 0.63), temperamental anger (OR: 0.72), and higher levels of support from family and friends (OR: 1.37).

To identify any interaction effects, we entered all two-way multiplicative interaction terms of predictors (between temperament and social support, between temperament and stressors, and between social support and stressors). No interaction effects were statistically significant, and their inclusion resulted in no changes in significance of main effects.

## Discussion

The aim of the current study was to expand knowledge of the role of stressors, social support and temperament measured early in child-rearing on developmental trajectories of maternal symptoms of depression and anxiety during the following 13 years of child rearing. To our knowledge, the current study is the first to examine the extent to which different types of stressors, social support and maternal temperament early in the child rearing phase can predict membership in groups based on maternal symptoms of depression and anxiety.

Analyses showed that each of these variables contributed to the differentiation of distinct trajectories, but with varying importance. We found that both risk and protective factors measured early in the child rearing period contributed to differentiation between symptom groups. This suggests that interventions directed to stressful early periods of parenting may be worthwhile in preventing symptoms of depression and anxiety among mothers. The main findings are further discussed below.

### Early stressors

The mean level of stressors at t1 was in general low among the mothers. However, we found that child related stressors and living condition related stressors at child age 18 months each were significant predictors of membership in groups with moderate or high levels of symptoms of depression and anxiety during the child-rearing period. Child related stressors early in the child rearing phase had a large effect. There was a twofold (OR: 2.16) increase in the odds of being in the High symptom group with an increase on the child related stress index. Hence problems regarding child care arrangements, combining work and family responsibilities, the child’s health and upbringing are important and they can be indicators of long term high levels of symptoms of depression and anxiety among mothers.

In accordance with findings from earlier mentioned studies [12,23], also lower education and being unemployed predicted membership in the High symptom group. Notably, being unemployed predicted a twofold increase in the risk of being in the High versus the Low symptom group. This shows the importance of socio-economic disadvantage, in addition to other stressors, in early childhood for trajectories of maternal symptoms of anxiety and depression from early childhood to adolescence. Many of the mothers in the current study had full-time paid jobs when the index child was 18 months. In addition to having paid work, women often do most of the child care and domestic work, even in gender equal countries like Norway [41]. Women often take care of both young children and for sick and elderly family members [17]. This role overload is believed to contribute to a sense of “burn out” and general distress, including depressive symptoms, in some women [17]. Different stressors might interact, and having high levels in one area might increase the experience of stress in another area. However, we found no significant interaction effects between the two sources of stress in our study. A possibility is that the sample was not big enough to discover significant interaction effects.

### Social support

A majority of the mothers (92%) had a partner when the child was 18 months, and reported that they received more support from their partners than from friends and family. Mothers in the Low-rising group reported the highest level of partner support when the target child was an infant, while the highest level of support from family and friends was reported among those in the Low symptom group. Mothers in the High symptom group reported the lowest level of support from both partners and from family/friends. A low level of support (of both types) was predictive of membership in the High symptom group in the multivariate analyses. Results from the analyses with the High symptom group as the reference group showed that partner support differentiated mothers in the Low-rising group from those in the High symptom group. Social support also differentiated mothers from the High and the Moderate symptom group, respectively, hence the less support the mothers reported when their children were infants, the more symptoms of depression and anxiety they experienced over time. Our findings support and expand upon the cross-sectional study by Mistry and colleagues (2007), who found that lack of emotional and functional social support for parenting increased the risk of poor mental health in mothers of children aged 4 to 35 months. Our findings replicate the findings from the field and provide evidence that lack of support early in the child rearing phase might have long-term implications.

In contrast to some studies that report that social support moderate the negative effects of stressors on symptoms of depression and anxiety [26,28], no interaction effects were found for support from either partner or friends and family. Our findings accord with other studies reporting relationships between social support and distress, regardless of the level of stressors [12,42]. The lack of interaction effects in our study could be explained by differences in effects of recent or short term stressors versus chronic stressors. Some findings suggest that social support only buffers the effects of recent stress, not long-term stress or adversities [26,43,44].

### Early maternal temperament

Mothers with a high stable trajectory of depression and anxiety across 13 years were significantly differentiated from mothers with stable low scores by higher levels on all three aspects of temperamental emotionality (i.e. distress, fearfulness and anger). Further, those with moderate levels of depression across time were significantly differentiated from those with stable low scores by higher levels of temperamental distress and fearfulness. The Low-rising group had higher levels of distress than the Low symptom group, and lower levels of anger than the High symptom group. The findings indicate that women with higher or increasing levels of symptomatology have the most negative temperament characteristics. Hence maternal temperament seems to be an important early indicator for symptom development and severity among mothers over the child rearing phase. The absence of interaction effects between temperament and the stressors suggests that its effects are independent of specific contexts of women’s lives.

There are several possible explanations for the importance of temperamental distress as a risk factor for high levels of symptoms of depression and anxiety. First, findings from other studies suggest that the genetically transmitted factor of neuroticism (which is closely related to temperamental emotionality) may predispose to both stressful life events and depression, or may increase the probability that an individual will respond to stress with depression [33]. Irrespective of the mechanism, it appears that temperament/personality gives important indications of vulnerability for symptoms of depression and anxiety.

A large proportion of mothers often experience lack of time, work overload, worries about childcare, and constant tiredness that affect their physical and mental well-being [45]. Our findings support other studies regarding work-family conflicts and well-being among mothers, which have found that work-family conflicts, because of problems in areas like combing work and child care arrangements, predict psychological distress [46,47].

### Strengths and limitations

Any investigation of the relationship of temperament and psychopathology is complicated by conceptual and methodological issues. Temperamental emotionality overlaps to some extent conceptually with symptoms of depression and anxiety, and associations can be artificially increased by overlapping item content. In this study, some of the items measuring distress (e.g. “I get emotionally upset easily”) and fearfulness (e.g. “I often feel insecure) are somewhat similar to HSCL items such as “feeling tense or keyed up” and “worrying too much about things”. Sanson, Prior and Kyrios (1990) first examined contamination between measures of child temperament and behaviour problems, showing that it was difficult to clearly distinguish between the two at a measurement level [48]. Later work, however, suggested that such confounding or overlap does not account for a large portion of the linkage between the two constructs [49,50]. This conclusion increases confidence that, while confounding may account for some of the associations in the current study, it does not provide a complete explanation of the associations found between temperament and symptom groups.

It could be argued that clinical diagnostic interviews of mothers would have been preferable to self reported symptoms. However, besides the non-feasibility of diagnostic interviews in large-scale studies, researchers have found self-reported symptom measures to be valid indicators when the purpose is to gain knowledge of high prevalent symptoms within a population [51]. An additional methodological issue is the use of self-ratings as the single source of information, where informant bias and distortions in self-image due to mood disturbance are possible. It might be that mothers who are experiencing symptoms of depression and anxiety see the world more negatively and that they therefore report more stressors and less social support (i.e. a depression-distortion effect). Despite the lack of viable alternatives for assessing these constructs in large-scale survey research, the potential for such bias should be taken into account in considering the results.

Internal consistency (Cronbach’s Alpha) was low for some of the predictor variables in the current study. In most cases, this was a result of the scales consisting of only three or four items covering a broad construct. The fact that we did find significant effects of the predictors suggests that they were not unreliable measures. However, the inclusion of such short scales may have lead to an underestimation of the effect sizes in the current study

The data in this study stem from a population-based sample which reduces the selection bias associated with studies of maternal symptomatology using clinical samples. Few studies of mental health have followed mother-child dyads over such a long time span as in this study. Despite attrition over time, the sample has remained reasonably representative of the Norwegian population, with relatively little bias. Nevertheless, the findings should be generalized to other populations only with care.

A strength of the current study was adopting a person-centred approach which made it possible to identify predictors for mothers with different symptom trajectories over time. The symptom trajectory groups used in the current study were derived by means of latent profile analysis (LPA) [24]. However, a limitation in the current multinomial logistic analyses was that, for simplicity, these groups (or pseudo-classes) were used instead of performing an analysis with the predictors in the LPA model. To include several predictors in a LPA model with data from six time points involves a complicated statistical model. In the approach used in the current study the uncertainty of latent class membership is not taken into consideration. However, the average posterior class probabilities for trajectory membership for our six group solution ranged from .80 to .91 hence the ambiguities regarding the class memberships are not large and the effect of not including the uncertainty of latent class membership in the analyses is not likely to produce substantial bias to the current results.

## Conclusions

This study showed that factors present early in the child rearing phase have substantial predictive power for explaining variance in maternal depressive symptoms over the following 13 years of child rearing. Temperamental distress and child related stressors were the strongest predictors of membership in different symptom trajectory groups. The persistence of symptoms over 13 years in the High symptom group suggests that it is important to be particularly aware of mothers with an accumulation of risk factors. From a public health perspective, these early indicators could inform prevention efforts. Living with symptoms of depression and anxiety throughout the child rearing phase is detrimental not only for the affected mothers, but also for their partners and children. Hence efforts directed towards minimising child related stressors and other risk factors early in the child rearing phase so as to prevent maternal symptoms of depression and anxiety deserve further attention.

## Competing interests

All authors declare that they have no competing interests.

## Authors’ contributions

AS performed the statistical analyses and drafted the manuscript. HJ has been involved in discussing the research questions, methods and analyses. KSM designed the project and collected the data. All authors were involved in discussing the idea and contributed to critically revise the manuscript. All authors read and approved the final manuscript.

## Pre-publication history

The pre-publication history for this paper can be accessed here:

http://www.biomedcentral.com/1471-2458/12/1120/prepub
